# Clusterin transcript variants expression in thyroid tumor: a potential marker of malignancy?

**DOI:** 10.1186/s12885-015-1348-0

**Published:** 2015-05-02

**Authors:** Paolo Fuzio, Anna Napoli, Anna Ciampolillo, Serafina Lattarulo, Angela Pezzolla, Nicoletta Nuzziello, Sabino Liuni, Francesco Giorgino, Eugenio Maiorano, Elda Perlino

**Affiliations:** 1 Institute of Biomedical Technologies, National Research Council (CNR), Via G. Amendola, 122/D, 70126 Bari, Italy; 2 Department of Emergency and Organ Transplantation, Section of Pathological Anatomy, University of Bari Aldo Moro, 70124 Bari, Italy; 3 Department of Emergency and Organ Transplantation, Section of Endocrinology, University of Bari Aldo Moro, 70124 Bari, Italy

**Keywords:** CLU, Gene expression, Thyroid tumour

## Abstract

**Background:**

Clusterin (CLU) is a ubiquitous multifunctional factor involved in neoplastic transformation. The CLU transcript variants and protein forms play a crucial role in balancing cells proliferation and death.

**Methods:**

We investigated the regulation of CLU transcript variants expression in an *in vivo* model system consisting of both neoplastic tissues and fine needle aspiration biopsy (FNAB) samples isolated from patients undergoing thyroidectomy.

**Results:**

The immunohistochemical analyses showed an overall CLU up-regulation in papillary carcinoma. A specific CLU2 transcript variant increase was registered using qPCR in papillary carcinomas while CLU1 decreased. In addition, the analysis of CLU transcripts expression level showed an increase of the CLU2 transcript in the TIR 3 patients with histologically confirmed thyroid cancer.

**Conclusions:**

Our results suggest the existence of a specific alteration of CLU2:CLU1 ratio towards CLU2, thus providing the first circumstantial evidence for the potential use of CLU transcript variants as effective biomarkers for a more accurate assessment of the so called “*indeterminate*” thyroid nodules.

## Background

Thyroid nodules are extremely common most of them are not cancerous, while malignant lesions derived from thyroid epithelial cells are relatively rare. The initial evaluation of thyroid nodules commonly involves thyroid function tests, ultrasound examination (USG) and fine needle aspiration biopsy (FNAB) of selected nodules. Clinically recognized thyroid carcinomas constitute 5-7% of all thyroid nodules and 1% of all human malignant tumours. The annual incidence of thyroid cancer varies worldwide from 0.5 to 10 per 100,000.

Thyroid carcinoma usually originates from follicular cells, and medullary carcinoma, which is a form of thyroid cancer, originates from the parafollicular C cells. Distinct histological types of follicular cell-derived cancers (FCDC) are recognized: the majority of cases are papillary, including its major subtype follicular variant (FVPTC); the remaining are follicular, oxyphilic or Hurthle cell, poorly differentiated and anaplastic carcinomas. Such tumour subtypes substantially differ from each other in terms of propensity to recurrence, distant spread and metastatic involvement [1,2].

Over the past 15 years, the application of molecular technologies to the study of these neoplasms has revealed critical genetic pathways associated with the development of specific thyroid tumour types [3]. The results obtained from several studies have shown that the progression from a single normal cell to a fully malignant phenotype requires both the activation of oncogenes and the inactivation of tumour suppressor genes.

Furthermore, signals from paracrine and/or autocrine pathways, alone or in combination, may regulate these processes, the final effects depending on the net balance between stimulatory and inhibitory factors. As the tumour progresses, neoplastic cells often lose the inhibitory growth factors in the regulation of cell proliferation, while the expression of positive autocrine growth factors increases. In this regard, the aberrant expression or function of regulatory genes, particularly of those encoding for adhesion factors, growth factors, apoptotic factors and their receptors, invariably occurs in several cancers, including thyroid carcinoma [4]. Among these, Clusterin (CLU) may represent a target gene that can be used to monitor changes that are responsible for the progression from normal to malignant and metastatic tissue.

CLU is a glycoprotein with a slightly ubiquitous tissue distribution and an apparent involvement in biological processes ranging from neurodegeneration in Alzheimer’s disease to cancer initiation and progression [5,6]. Indeed, CLU has been implicated in numerous physiologic and pathologic processes important for carcinogenesis and tumour growth, including apoptotic cell death, cell cycle regulation, DNA repair, cell adhesion, tissue remodeling, lipid transportation, membrane recycling and immune system regulation [5,6]. Understanding the different functions of CLU has been an elusive goal, as it its processes are of very different nature and sometimes contradictory. This ambiguity is partly due to the existence of two functionally divergent protein forms: a glycosylated secreted heterodimer of approximately 80 kDa (sCLU) and a 55 kDa non-glycosylated nuclear form (nCLU). sCLU is the secreted heterodimer with documented anti-apoptotic function, while nCLU was reported to move from the cytoplasm to the nucleus following certain cytotoxic events and postulated to induce apoptosis [7]. The nCLU protein form can be synthesized from a second in-frame AUG codon and does not undergo cleavage or extensive glycosylation. Recent studies provide strong evidence for an anti-apoptotic function for sCLU [8], even though precise site(s) of action and binding proteins remain poorly defined. This study suggested that elevated levels of CLU in several human cancers might promote tumour progression by interfering with pro-apoptotic pathways by interaction with activated Bax, thereby inhibiting cytochrome C release and apoptosis [8]. The biology of CLU is however even more complex. Indeed, analogous to the two forms of Bcl-x that arise from alternative splicing, with the long form having anti-apoptotic and the short form having pro-apoptotic properties, the mature sCLU has a cytoprotective function, while under certain conditions pro-apoptotic signals may induce the expression of the nCLU protein forms [9,10].

Recent studies have demonstrated there is no relationship between CLU transcript variants and CLU protein forms, showing as CLU1 and CLU2 code for the same secreted protein (sCLU) also identical regarding the post-transcriptional modifications (i.e. glycosylation) [11,12]. However, the characterization of CLU and its functional role have not been clearly established yet.

Increased expression of CLU has been found in different diseases where either abnormal cell death or proliferation occurs [13]. Moreover, CLU mRNA and protein were found over-expressed in several human cancers such as prostate, breast, lung, kidney, ovarian, colon, and endometrial tissues [14-22]. These findings indicate that CLU is a cell survival gene up-regulated by apoptotic triggers, and when over-expressed may confer resistance to apoptosis, representing a potential therapeutic target for cancer. In preclinical models of prostate cancer, in fact, CLU antisense oligonucleotides improved the efficacy of chemotherapy, radiation therapy, and androgen withdrawal by enhancing the apoptotic response [23].

CLU was identified by microsequencing in the culture medium of porcine thyrocytes [24]. Treatment of thyrocytes with thyroid stimulating hormone (TSH) revealed a tight regulation of both synthesis and secretion of CLU, with a distinct fraction of CLU being always associated with the cells. The association with the apical plasma membrane, which carries the iodinating system in thyrocytes, was confirmed by biosynthetic iodination and CLU was found within distinct, bipartite patches, suggesting that it is a constituent of cell-adhesion complexes and participates in cell-cell and cell-matrix interactions [24].

A large number of genes, which are regulated by the thyroid, can be potentially used as marker genes for cancer diagnosis and prognosis. Previous data suggested that CLU expression in thyroid malignant cells was modified *in vitro* and *in vivo*, and that there was a complex mechanism of regulation of CLU expression in the normal and cancerous thyroid tissue [25,26].

Changes in CLU expression may thus play a role in the pathogenesis of the thyroid malignant transformation. In this light, we measured the CLU expression at mRNA and protein level in neoplastic and non-neoplastic thyroid tissues, to assess the potential role of CLU as a biomarker for thyroid cancer.

Moreover, we investigated potential changes in CLU expression in fine needle aspiration biopsy (FNAB) of thyroid nodules with indeterminate (TIR 3) cytology to assess the potential use of CLU as a biomarker to discriminate between benign and malignant lesions, thus obtaining relevant information to select the patients to submit to thyroidectomy for malignancy.

## Results

### CLU immunohistochemistry

CLU immunoreactivity was evaluated in 50 thyroid tissues isolated from patients with adenoma (n = 40) and papillary carcinoma (n = 10).

**Figure 1:**
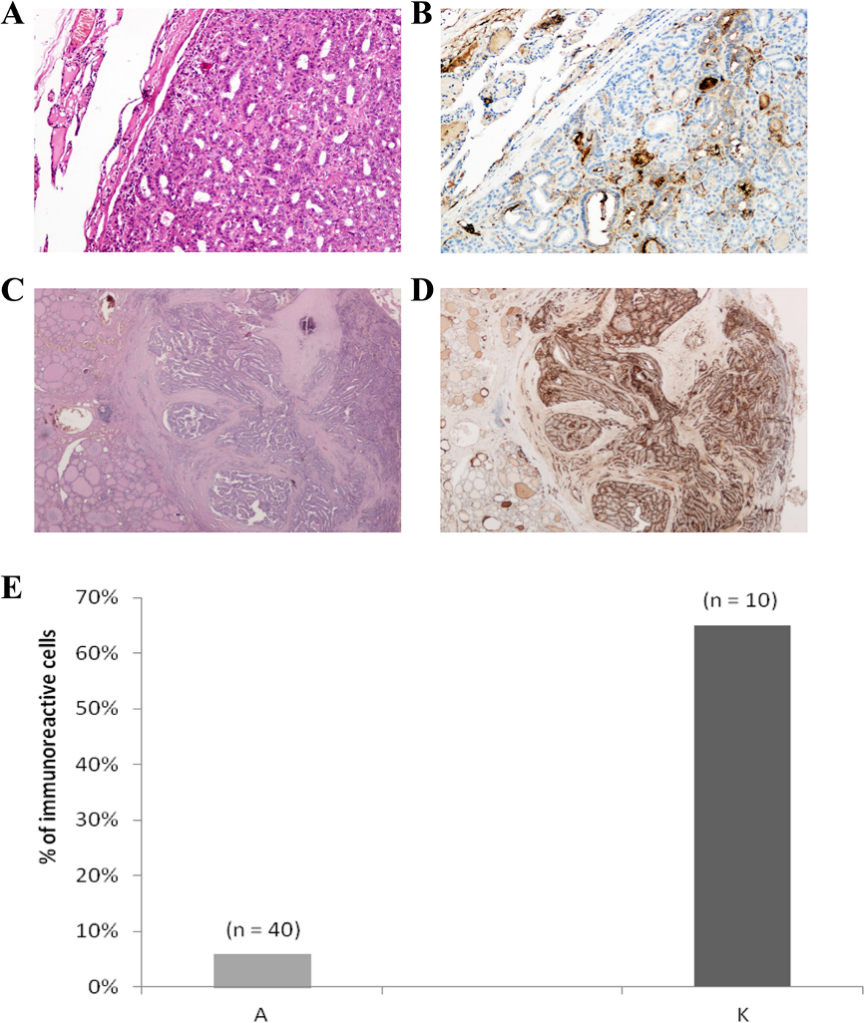
CLU immunohistochemical analyses. **(A)** Hematoxylin-Eosin staining of a follicular adenoma. **(B)** Anti-CLU antibody immunostaining of the same adenoma. **(C)** Hematoxylin-Eosin staining of a papillary carcinoma with adjacent normal thyroid tissue. **(D)** Anti-CLU antibody immunostaining of the same carcinoma. **(E)** Immunoreactive epithelial cells in thyroid carcinomas (K, n = 10) and thyroid adenomas (A, n = 40) samples, respectively.

Figure 1 shows the Hematoxylin-Eosin and the immunohistochemical microscopic features of a follicular adenoma (A and B, respectively) and a papillary carcinoma (C, D). Anti-CLU antibody clearly highlights positive staining in the cytoplasm of thyrocytes in all sections, according to previously reported findings on CLU immunoreactivity in breast, prostate and ovarian tissues [14,18,19].

Figure 1E shows the percentage of CLU immunoreactive cells in thyroid adenomas (A) and papillary carcinomas (K) respectively. The average percentage of CLU-immunoreactive cells was much higher (almost 6 fold) in papillary thyroid carcinomas than in thyroid adenomas. Comparable results were obtained with both the antibodies used, with less than 5% variation in the percentage of immunoreactive cells of each sample. These results suggest that CLU-immunoreactivity is detectable in a much higher number of neoplastic cells in papillary thyroid carcinomas in comparison with adenomas.

### Analysis of CLU mRNA variants

Since antibodies specific to each of the CLU protein forms are not available, we could not evaluate the different protein CLU variants but the α subunit of the CLU heterodimer was recognized by the (Clone 41D) antibody that is a monoclonal anti-human CLU. In this light, the different CLU transcript variants expressed in the thyroid tissues were evaluated by qPCR. To identify all possible novel CLU transcript variants, we first inspected the CLU entries in the ASPicDB, the Alternative Splicing Prediction Data Base (http://srv00.ibbe.cnr.it/ASPicDB/) [27,28]. These inspections revealed multiple possible CLU transcript variants, two of which identified by the Signature ID [29] c7175b345e:9 and 1057fea355:9, overlapping to the sequences of the NCBI database with accession number NM_001831, CLU1 and NR_038335, CLU2. These two variants all contain nine exons, and each of them presents a unique exon 1 and shares the remaining 2–9 exons [21]. Although the CLU1 transcript variant was not present in other cancer tissues [11,30] the reason why it is taken into consideration is because it stood out by having substantially more sequence support than the others in the ASPicDB *in silico* analysis.

By RT-PCR analysis followed by sequencing experiments, the two CLU transcript variants were found to be expressed in the human thyroid tissues. Moreover, by using the specific CLU primers [21], no additional bands were detected except the two variant-specific RT-PCRs (data not shown).

Moreover, potential quantitative changes in CLU transcript variants expression were investigated by means of qPCR as reported in Materials and Methods in the thyroid tumour tissues in comparison to the corresponding normal tissues isolated from the same patients (see Table 1). Raw fluorescence data were normalized with respect to the GAPDH expression level in all samples and the CLU transcript variants expression obtained in thyroid tumour samples was normalized to the corresponding normal samples.

**Table 1:** **Clinical features of patients affected by thyroid carcinoma**

Patients	Age (years)	Gender	Histology	FNAB classification (SIAPEC)	TNM Stage
N1	26	Female	Normal Thyroid	-	-
K1			Papillary Thyroid Carcinoma	TIR5	T3NxMx
N2	60	Male	Normal Thyroid	-	-
K2			Papillary Thyroid Carcinoma	TIR4	T1bNxMx
N3	58	Female	Normal Thyroid	-	-
K3			Papillary Thyroid Carcinoma	TIR5	T3NxMx
N4	60	Male	Normal Thyroid	-	-
K4			Papillary Thyroid Carcinoma	TIR5	T2bNxMx
N5	37	Male	Normal Thyroid	-	-
K5			Follicular Thyroid Carcinoma	TIR3	T3NxMx
N6	58	Male	Normal Thyroid	-	-
K6			Follicular Thyroid Carcinoma	TIR4	T4aNxMx
N7	-	Female	Normal Thyroid	-	-
K7			Papillary Thyroid Carcinoma	TIR3	T1bNxMx
N8	-	Female	Normal Thyroid	-	-
K8			Papillary Thyroid Carcinoma	TIR4	T2bNxMx
N9	-	Female	Normal Thyroid	-	-
K9			Follicular Thyroid Carcinoma	TIR3	T1bNxMx
N10	-	Female	Normal Thyroid	-	-
K10			Follicular Thyroid Carcinoma	TIR3	T2bNxMx
N11	38	Female	Normal Thyroid	-	-
K11			Thyroid Adenoma	TIR3	-
N12	50	Female	Normal Thyroid	-	-
K12			Thyroid Adenoma	TIR3	-
N13	72	Male	Normal Thyroid	-	-
K13			Thyroid Adenoma	TIR3	-
N14	79	Female	Normal Thyroid	-	-
K14			Thyroid Adenoma	TIR4	-
N55	-	Female	Normal Thyroid	-	-
K15			Thyroid Adenoma	TIR3	-

- : data unknown.

**Figure 2:**
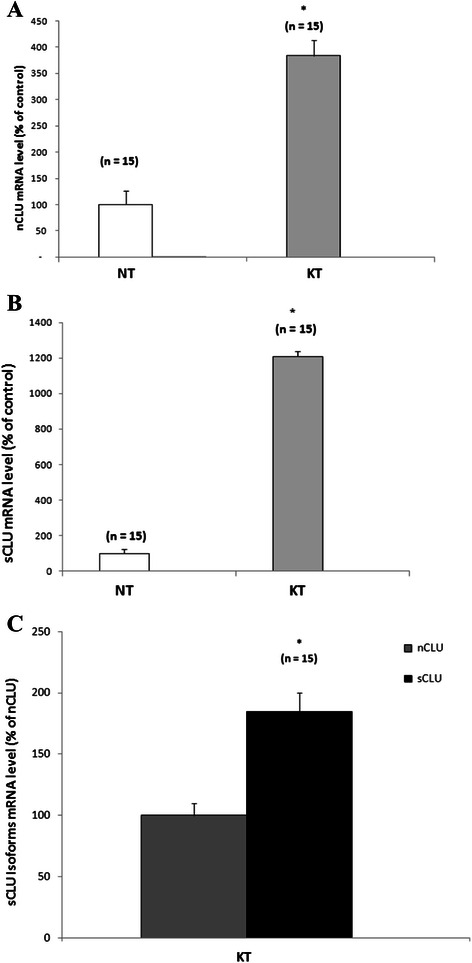
CLU mRNA variants analyses. CLU transcript variants levels of malignant tissues (KT) compared to normal thyroid (NT). **(A)** CLU1 expression level. **(B)** CLU2 expression level. **(C)** CLU2 expression compared to CLU1, arbitrarily setted as 100%.

The CLU1 expression level was almost 4-fold higher in the thyroid cancer tissues than in the normal thyroid tissues (Figure 2A). The CLU2 expression level was also markedly elevated (10-fold) in the thyroid cancer tissues in comparison to the normal thyroid tissues (Figure 2B).

Since it has been suggested that the CLU2:CLU1 cellular balance may be critical for cancer development and progression [31], the analysis of the ratio of the two transcript variants was investigated. The relative concentration of CLU2:CLU1 transcript variants in tumour samples shows a down regulation of the CLU1 expression. Using the CLU1 level as calibrator arbitrarily set as 100%, the CLU2 expression resulted higher (185% ± 15) than CLU1 (Figure 2C).

Even though the statistical analysis is not sufficiently reliable, due to the limited number of investigated cases with different TNM staging (T1, n = 3; T2, n = 3; T3, n = 3; T4, n = 1), findings (data not shown) suggest a potential shift in the CLU transcript variants expression from the pro-apoptotic CLU1 protein forms to the cytoprotective-oncogenic CLU2 protein form during the transition from normal to malignant cells.

### CLU transcript variants expression in the indeterminate thyroid nodules

**Figure 3:**
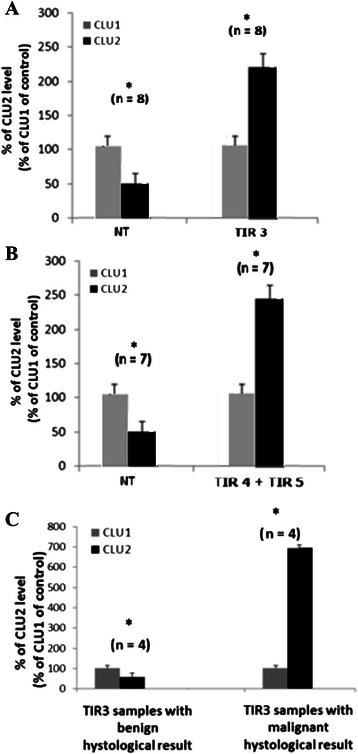
CLU mRNA variants analyses according to the SIAPEC cytological classification. CLU2 and CLU1 expression in the TIR3 **(A)** and TIR4 + TIR5 **(B)** neoplastic thyroid samples, compared to normal thyroid tissues (NT). Data are expressed according to the histological diagnosis after surgery. **(C)** CLU2 expression in TIR3 thyroid samples according to histological classification (i.e., benign vs. malignant) after surgery. CLU2 expression was compared to CLU1, arbitrarily setted as 100%.

The absence of biomarkers which could be used in case of indeterminate tumors (i.e., TIR3) to support the clinician’s decision to recommend surgery, represents one of the most debated issues in the management of thyroid nodules. In this light, we investigated the balance between the two CLU transcript variants in thyroid nodules with different cytological diagnosis following fine needle aspiration biopsy (FNAB) (see Table 1), and specifically TIR 3 (indeterminate), TIR 4 (suspicious) and TIR 5 (malignant), according to the SIAPEC cytological classification [32]. We also compared the results to the histological diagnosis after surgery, to assess whether the lesions were benign or malignant. Regardless of the benign or malignant nature, after the histological test, CLU2 expression was always higher than CLU1 in all TIR 3 (242 ± 25%) and TIR 4 + TIR 5 (233 ± 21%) malignant thyroid tissues samples (Figures 3A and B) and this increase was always statistically significant (p < 0.05). Our results show a shift of the CLU2:CLU1 ratio in favour of CLU2 in both TIR3 and TIR4 + TIR5 cytological samples with a histologically proved diagnosis of malignancy.

Figure 3C shows CLU2 expression levels measured in TIR3 samples, according to histological classification obtained after surgery (i.e., benign vs. malignant) in comparison to the CLU1 expression used as control. Histology indicated a benign lesion in 4/8 of the TIR3 samples and a malignant lesion in the remaining 4 samples. In the benign TIR3 thyroid tissues, CLU2 expression levels were lower than CLU1 level (73 ± 20%, p < 0.05). On the contrary, in the malignant TIR3 thyroid tissues, CLU2 expression levels were higher than CLU1 levels (410 ± 30%, p < 0.05).

In conclusion, these results show an increase of CLU2 in malignant thyroid tissues, suggesting a possible use of this transcript variant to discriminate between those TIR3 (*indeterminate*) cases proven malignant at histological examination and those diagnosed as benign nodules.

### CLU transcript variants expression in thyroid fine-needle aspiration

Finally, we measured the transcript levels of the two CLU transcript variants in the FNAB samples of 15 patients (K1-K15) with indeterminate thyroid nodules cytology (see Table 1) to verify the potential diagnostic use of CLU to pre-operatively discriminate among patients undergone a thyroidectomy for malignancy. In the FNABs samples CLU2 expression was higher than CLU1 (185% ± 15; p < 0.05; data not shown) as already shown in thyroid tissues.

In the TIR3 and TIR4 + TIR5 FNAB cytological samples, CLU2 expression levels were higher than CLU1 (190% ± 10 and 175% ± 12, respectively; p < 0.05; data not shown) suggesting a shift in the CLU2:CLU1 ratio in favour of the CLU2 transcript variant, thus confirming the results obtained in the thyroid tissues.

**Figure 4:**
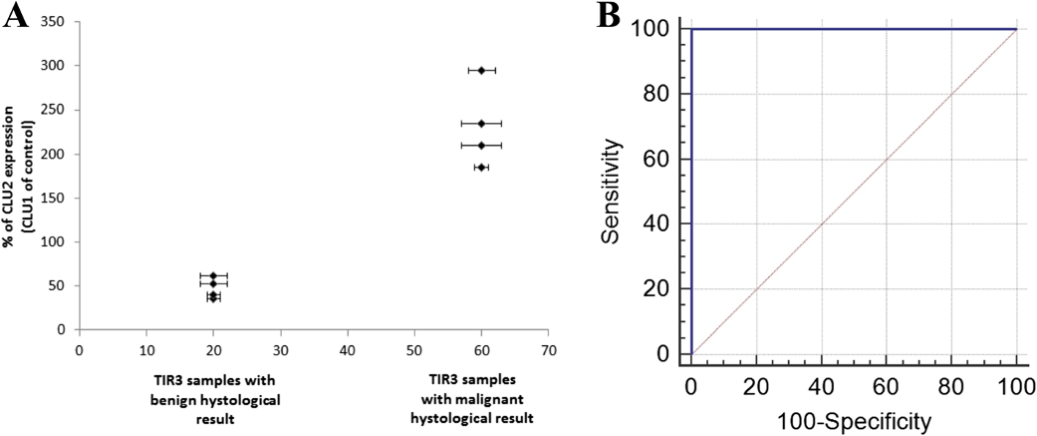
CLU2 expression in the TIR3 FNAB samples classified according to the histopathological diagnosis. **(A)** CLU2 expression was compared to CLU1, arbitrarily setted as 100%. **(B)** ROC curve analysis showed the usefulness of the ratio of CLU2:CLU1 expression for clearly distinguishing the malignant thyorid tumors from adenoma in patients with TIR3 thyorid FNAB lesions. MedCalc statistical software was used for statistical analysis.

The CLU transcript variants expression level was also measured in TIR3 samples classified according to their benign or malignant nature, as determined by histopathological examination (Figure 4). In histologically benign TIR3 FNABs, the CLU2 transcripts level was always lower than CLU1 transcripts level (ranging from 31 ± 7% to 81 ± 8%; average value = 47 ± 10%; p < 0.05). On the contrary, in histologically malignant TIR3 FNABs, CLU2 levels were higher than CLU1 levels (ranging from 175 ± 11% to 306 ± 12%; average value = 229 ± 20%; p <0.05).

The usefulness of the ratio of CLU2:CLU1 expression to distinguish the malignant thyroid tumors from adenoma in patients with TIR3 thyroid FNAB lesions was evaluated using receiver operating curve (ROC) analysis performed by MedCalc Statistical Software. Although our results will be verified on a larger population, the AUC was 1.00 with 100% specificity and 100% sensitivity (Figure 4B).

In conclusion, our results suggest a specific increase of CLU2 in TIR3 thyroid FNAB lesions which were found to be malignant at histological examination, suggesting a possible role of the CLU2 transcript variant to discriminate between those cytologically indeterminate (TIR3) lesions that were histologically diagnosed as benign or malignant, thus potentially supporting the clinician’s decision to operate on these patients.

## Discussion

Although several molecules are associated with thyroid carcinogenesis, their role in malignant transformation has not been well established yet. Over the past 15 years, the application of molecular technologies to the study of this neoplasm has revealed critical genetic pathways associated with the development of specific thyroid tumour subtypes.

Despite the growing evidence of the role of CLU in the aetiology of cancer in several tissues, the regulation of its expression and functions in human thyroid carcinoma has not been investigated.

As already mentioned, CLU is expressed by thyrocytes and undergoes a TSH-dependent regulation [24]. Furthermore, CLU may influence cellular apoptosis in neoplastic cells, as recently shown in retinoblastoma cells, in which overexpression of CLU was associated with enhanced cellular apoptosis [33].

In order to assess the possible role of this gene as a candidate biomarker for thyroid cancer, we investigated CLU transcript variants in neoplastic and non-neoplastic thyroid tissues by measuring protein and transcript expression by immunohistochemical and qPCR techniques.

Immunohistochemical analysis showed an overall up-regulation of CLU protein expression in papillary carcinoma. The prevalent CLU protein expression in the thyroid carcinoma, in agreement with results from other laboratories, suggests that CLU may play a cytoprotective role in mammalian epithelia, including thyroid, oesophagus, urethra and rete testis epithelial cells [3]. The results from this study are also in agreement with the findings showing increased CLU transcript variants expression in hormonally regulated human cancers, such as prostate and breast carcinomas [10,14]. In both cases, CLU up-regulation was associated with increased cancer cell survival, suggesting that CLU over-expression may contribute to increased tumour cell survival. We found here for the first time that the two CLU transcript variants are expressed in thyroid tissues and that CLU transcript expression is up-regulated *in vivo*.

The CLU1 expression in thyroid cancer tissues was almost 4-fold higher than in normal thyroid. Furthermore, CLU2 transcripts expression level resulted markedly increased (12-fold) in thyroid cancer tissues, in comparison with normal thyroid tissues.

Since it has been suggested that the CLU2:CLU1 cellular balance may be critical for cancer initiation and progression, an analysis on the ratio of transcripts level encoding each of the two CLU1 and CLU2 variants in the thyroid tissues was carried out. This is the first time that statistically significant variations in the CLU2:CLU1 ratio in papillary carcinoma have been found, confirming similar findings in other tumors, such as prostate [18], breast [14], lung [17], colon [16], and ovary [19] cancers, thus suggesting that this shift toward increased CLU2 may mark the transition from the normal to the neoplastic cell phenotype. Specifically, the neoplastic transformation may manifest itself with the alteration of synthesis of the two CLU transcript variants with a dominance of CLU2 over CLU1, resulting in the promotion of transformed cell survival. This hypothesis is in agreement with previous studies suggesting that the survival of cancer cells is related to over-expression of CLU2 and down-regulation of CLU1 transcripts [21].

One of the most debated issues in the management of thyroid nodules is the absence of reliable biomarkers that can effectively help to identify, among patients carrying cytologically indeterminate (TIR3) nodules, those who necessitate further treatment due to the malignant nature of their lesion. Currently, the management of patients carrying thyroid nodules with indeterminate cytology is limited by the intrinsic limitations of FNAB, and in such cases the occurrence of abnormal cellular features preclude a definitive assessment of benignity, although only a minority will prove malignant upon final histopathological examination. Consequently, patients with indeterminate cytopathological reports are commonly referred for consideration of hemi- or near-total thyroidectomy. We attempted to address the potential usefulness of CLU transcript variants in this context by measuring the balance between CLU2 and CLU1 in different cytological categories corresponding to TIR3 and TIR4 + TIR5 samples, classified according to the SIAPEC. The analysis of CLU transcript variants expression showed a specific increase of the CLU2 in TIR3 patients with histologically proven thyroid cancer, suggesting a possible diagnostic use of CLU2 in the management of such patients.

The possible role of CLU in thyroid tumours prognosis would give more relevance to the results of our paper. Nevertheless, it is well known that thyroid tumours, especially the well differentiated types (which are the majority of our cases) follow a very indolent course and though we detected a limited number of lymph node metastases, their number was not at all adequate to draw any significant conclusion. The same also applies to tumour recurrence, which, though present in our series, were too limited in number to allow statistical analyses and, above all, seemed more strictly related to tumour size and extra capsular spread than to Clusterin expression. Considering this, we decided to skip the analysis of Clusterin as a potential prognostic marker and focus our investigation on its role as a diagnostic aid.

## Conclusions

Despite the need to further substantiate these results in a larger and more representative cohort, and albeit the conclusion are still speculative, they provide new insights into the role of CLU as a pro-survival gene that is up-regulated in thyroid cancer. We hope this change may have possible implications for the management of thyroid cancer to better identify cytological indeterminate lesions to be treated with surgery.

## Methods

### Patients

This study was carried out in the University Hospital Policlinico (Bari, Italy) using 65 thyroid tissue specimens from total thyroidectomies from patients harbouring lesions that were 1 cm or more, to make sure that enough pathological tissue was left from each patient for subsequent pathological reporting.

We obtained the participants’ informed consent form approved by the local ethical committees (AOU Policlinico, 70124 Bari, Italy) for their participation and for publication of the dataset collected at recruitment, in compliance with international and national data protection laws. The dataset is fully anonymous, as it does not contain any direct or indirect identifier, thus respecting participants’ rights to privacy and identity protection.

Among the 65 total tissues, 50 histological embedded paraffin thyroid tissues (40 thyroid adenomas, 2 microcarcinomas, measuring less than 1 cm, and 8 papillary carcinomas, collected from the database of the Department of Pathology of the University of Bari) were investigated by immunohistochemistry.

The remaining 15 thyroid tissues (Table 1) were prospectically collected after thyroid surgical resection and immediately frozen and/or stored at −80°C. All patients underwent FNAB before surgery. Routinely, each surgical sample is subjected to intra-operative (i.e., frozen section) examination to confirm the neoplastic nature of the lesion and assess the presence of normal adjacent tissues. Based on the results of intra-operative examination, the pathologist can obtain fresh samples from both neoplastic and normal tissues to be used for molecular analyses. For each patient, biopsy specimens of normal (N1-N15) and pathological thyroid (K1-K15) tissues, were collected and analysed. TNM stage [34] and the cytological classification according to SIAPEC (Italian Society Anatomy Pathology and Cytology) [32] are reported. According to the British Thyroid Association, the AACE/AME Task Force on Thyroid Nodules, the Italian Consensus Working Group and the Bethesda system for Thyroid Cytopathology, inadequate sampling was classified as TIR1; benign lesions were categorized as TIR2; neoplastic/proliferative lesions, characterized by distinct follicular aggregates without obvious cytological atypia, as TIR3; lesions that were suspicious for malignancy as TIR4; frankly malignant lesions as TIR5.

As reported in Table 1, 10 patients were affected by thyroid carcinoma, 4 of the follicular subtype (K5, K6, K9, K10) and 6 papillary carcinomas (K1-K4, K7-K8) (median age at surgery: 47 ± 5 years). Their FNAB yielded the following results: TIR5, n = 3; TIR4, n = 3; TIR3, n = 4. The remaining 5 patients (K11-K15), were classified as TIR4 (n = 1) and TIR3 (n = 4) after FNAB and were affected by non-neoplastic thyroid disease, as proven at subsequent histological examination.

### Histopathology and immunohistochemistry

The surgical samples were fixed in 10% neutral buffered formalin for 12–24 hours, embedded in paraffin, cut and stained with Hematoxylin-Eosin.

From the selected cases for which sufficient and representative amounts of tissues were available after morphological analysis, a single paraffin block was selected for immunostaining. Five-μm thick sections were cut, collected on positively charged slides, de-waxed and re-hydrated. Following quenching of endogenous peroxidase with 3% H_2_O_2_ for 15 minutes at room temperature, the sections were immunostained for CLU using a peroxidase-based detection system (En Vision, Dako, Glostrup, Denmark) with an automated immunostainer (Autostainer, Dako). Prior to the staining procedure, the sections to be incubated with anti-CLU antibody were immersed in 0.01 M citrate buffer, pH 6.0, and incubated at 98°C for 30 minutes in a water bath. Two distinct mouse monoclonal antibodies identifying α-CLU were used in this study: clone 41D (provided by Upstate, Lake Placid, NJ, USA, dilution 1:200) and clone B-5 (provided by Santa Cruz Biotechnology, Santa Cruz, CA, USA, dilution 1:100) both with 20 minutes incubations at 37°.

Control sections for antibody specificity included staining of positive (normal and carcinomatous prostate) as well as negative sections, which were incubated with the immunoglobulin fraction of normal mouse serum.

In all cases, the immunoreactivity was independently evaluated by two pathologists (E. Maiorano and A. Napoli) by separately counting the relative number of immunoreactive epithelial cells in 10 different microscopic fields of each case, observed at 40x magnification.

### RNA extraction and qPCR experiments

Frozen tissue samples were pulverized and cellular RNA was extracted using the Trizol procedure, as previously described [21]. Quantification of RNA was carried out using NanoDrop ND-1000 (Thermo Scientific, Wilmington, DE, USA) spectrophotometer; the quality of RNA was evaluated on Agilent 2100 Bioanalyzer (Agilent Technologies, Santa Clara, CA, USA) using a RNA 6000 Nano chip kit, RNA ladder and Agilent analysis software (Agilent Technologies).

cDNA synthesis was performed from 1 μg of total RNA using the QuantiTect® Reverse Transcription kit (Qiagen). Variant-specific PCRs, using the cDNAs as templates, were performed using the GoTaq® Flexi DNA Polymerase (Promega). Variant-specific primer pairs were constructed with Primer3 software, combining specific forward primers located in the unique exons 1 (1a, 1b) annealing downstream of the ATG and the reverse primer located in exon 2. The primer sequences have been previously listed [21]. Note that the CLU1 amplicon obtained contains the TATA box sequence suggested by other authors [11,30] not present in prostate and colon cancer.

1 μl of each cDNA was used as template in qPCR assays, performed in triplicate with ABI PRISM 7900HT (Applied Biosystems) using the QuantiTect® SYBR Green PCR Master Mix (Qiagen). Amplification parameters have been previously listed [21]. Fluorescence raw data were exported from the SDS 2.2.1 software (Applied Biosystems) and analysed with the DART-PCR Excel workbook [35]. For each tissue, the relative expression ratio (rER) of different CLU transcripts was calculated by applying the following formula: [(1 + E_(calibrator)_)^Ct_(calibrator)_/ (1 + E_(target)_)^Ct_(target)_], where E is the average amplification efficiency calculated by DART-PCR for each primers pair and Ct is the average Ct obtained for the calibrator (normal thyroid tissues) and for the target (all thyroid tissue analysed). Moreover, for each CLU transcript variants the relative expression ratio (rER) with respect to the analysed thyroid tissue was calculated by applying the following formula: [(1 + E_(target)_)^(Ct_sample_ – Ct_calibrator_)_target_]/[(1 + E_(EC)_) (Ct_sample_ – Ct_calibrator_)_EC_], where EC is the endogenous control and CLU1 the calibrator. The average data from at least two independent experiments are reported.

### Statistical analysis

Data are reported as the mean ± SEM. Statistical analysis was performed using the Student’s t-test.
